# (*E*)-*N*′-(3,4-Di­meth­oxy­benzyl­idene)nicotinohydrazide monohydrate

**DOI:** 10.1107/S1600536814013798

**Published:** 2014-06-18

**Authors:** J. Josephine Novina, G. Vasuki, M. Suresh, M. Syed Ali Padusha

**Affiliations:** a Department of Physics, Idhaya College for Women, Kumbakonam-1, India; b Department of Physics, Kunthavai Naachiar Govt. Arts College (W) (Autonomous), Thanjavur-7, India; c PG & Research Department of Chemistry, Jamal Mohamed College (Autonomous), Tiruchirappalli-20, India

## Abstract

In the title hydrated compound, C_15_H_15_N_3_O_3_·H_2_O, the nicotinohydrazide mol­ecule adopts a *trans* conformation with respect to the C=N double bond. The dihedral angle between the benzene and pyridine rings is 5.10 (14)°. In the crystal, the solvent water mol­ecule acts as an acceptor, forming an N—H⋯O hydrogen bond supported by two C—H⋯O contacts. It also acts as a donor, forming bifurcated O—H⋯(O,O) and O—H⋯N hydrogen bonds that combine with the former contacts to form zigzag chains of mol­ecules along the *c*-axis direction. An additional O—H⋯O donor contact completes a set of six hydrogen bonds to and from the water mol­ecule and connects it to a third nicotinohydrazide mol­ecule. This latter contact combines with weaker C—H⋯O hydrogen bonds supported by a C—H⋯π contact to stack mol­ecules along *b* in a three-dimensional network.

## Related literature   

For the biological activity of hydrazone compounds, see: Singh & Raghav (2011 ▶); Patil *et al.* (2011 ▶). For background to the use of nicotinohydrazides as catalysts and of their transition metal complexes in the treatment of tuberculosis, see: Torje *et al.* (2012 ▶). For closely related structures, see: Novina *et al.* (2013 ▶); Wang *et al.* (2010 ▶).
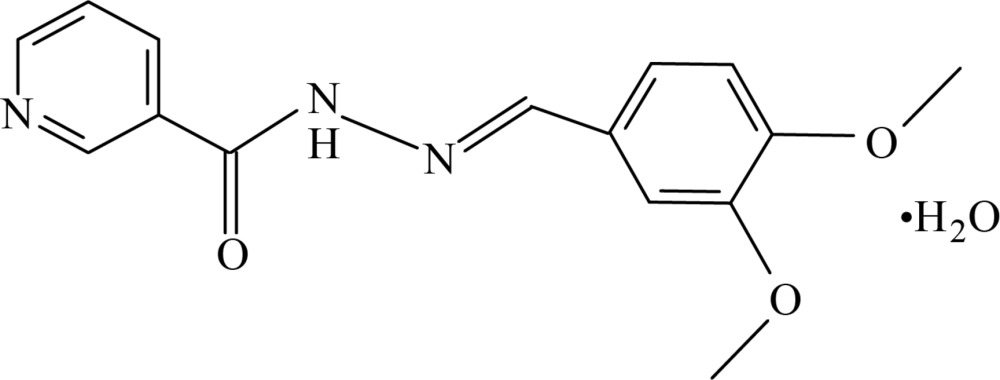


## Experimental   

### 

#### Crystal data  

C_15_H_15_N_3_O_3_·H_2_O
*M* *_r_* = 303.32
Monoclinic, 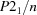

*a* = 4.9128 (6) Å
*b* = 25.137 (4) Å
*c* = 12.2950 (16) Å
β = 96.513 (4)°
*V* = 1508.6 (4) Å^3^
*Z* = 4
Mo *K*α radiation
μ = 0.10 mm^−1^
*T* = 296 K
0.50 × 0.35 × 0.30 mm

#### Data collection  

Bruker Kappa APEXII CCD diffractometer
Absorption correction: multi-scan (*SADABS*; Bruker, 2004 ▶) *T*_min_ = 0.952, *T*_max_ = 0.971
11633 measured reflections
3704 independent reflections
2250 reflections with *I* > 2σ(*I*)
*R*_int_ = 0.036

#### Refinement  

*R*[*F* ^2^ > 2σ(*F* ^2^)] = 0.048
*wR*(*F* ^2^) = 0.141
*S* = 1.02
3704 reflections
209 parameters
3 restraints
H atoms treated by a mixture of independent and constrained refinement
Δρ_max_ = 0.21 e Å^−3^
Δρ_min_ = −0.23 e Å^−3^

### 

Data collection: *APEX2* (Bruker, 2004 ▶); cell refinement: *APEX2* and *SAINT* (Bruker, 2004 ▶); data reduction: *SAINT* and *XPREP* (Bruker, 2004 ▶); program(s) used to solve structure: *SIR92* (Altomare *et al.*, 1993 ▶); program(s) used to refine structure: *SHELXL97* (Sheldrick, 2008 ▶); molecular graphics: *ORTEP-3 for Windows* (Farrugia, 2012 ▶) and *Mercury* (Macrae *et al.*, 2008 ▶); software used to prepare material for publication: *PLATON* (Spek, 2009 ▶).

## Supplementary Material

Crystal structure: contains datablock(s) I, global. DOI: 10.1107/S1600536814013798/sj5412sup1.cif

Structure factors: contains datablock(s) I. DOI: 10.1107/S1600536814013798/sj5412Isup2.hkl

Supporting information file. DOI: 10.1107/S1600536814013798/sj5412Isup3.cml

CCDC reference: 1008073

Additional supporting information:  crystallographic information; 3D view; checkCIF report

## Figures and Tables

**Table 1:** Hydrogen-bond geometry (Å, °) *Cg*2 is the centroid of the C2–C7 benzene ring.

*D*—H⋯*A*	*D*—H	H⋯*A*	*D*⋯*A*	*D*—H⋯*A*
N2—H2*N*2⋯O1*W* ^i^	0.86	2.06	2.8942 (19)	165
O1*W*—H2*O*1⋯O3	0.85 (2)	2.15 (2)	2.955 (2)	157 (3)
O1*W*—H2*O*1⋯N1	0.85 (2)	2.49 (2)	3.1087 (19)	130 (2)
C11—H11⋯O1*W* ^i^	0.93	2.30	3.199 (3)	162
C8—H8⋯O1*W* ^i^	0.93	2.67	3.425 (2)	139
O1*W*—H1*O*1⋯O3^ii^	0.86 (2)	2.09 (2)	2.901 (2)	159 (3)
C1—H1*C*⋯*Cg*2^ii^	0.96	2.88	3.729 (3)	148

Symmetry codes: (i) 
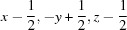
; (ii) 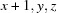.

**Fig. 1.**
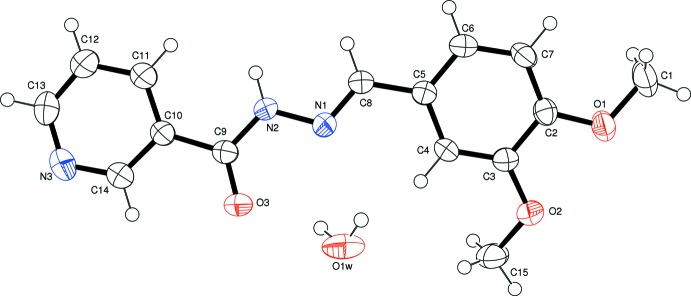
The molecular structure of the title compound, with the atom labelling. Displacement ellipsoids are drawn at the 50% probability level.

**Fig. 2.**
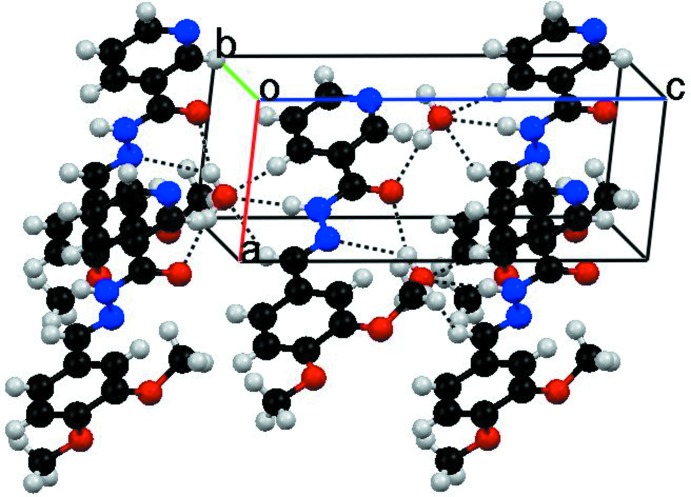
Crystal packing of the title compound viewed along the b axis. Hydrogen bonds are shown as dashed lines.

**Fig. 3.**
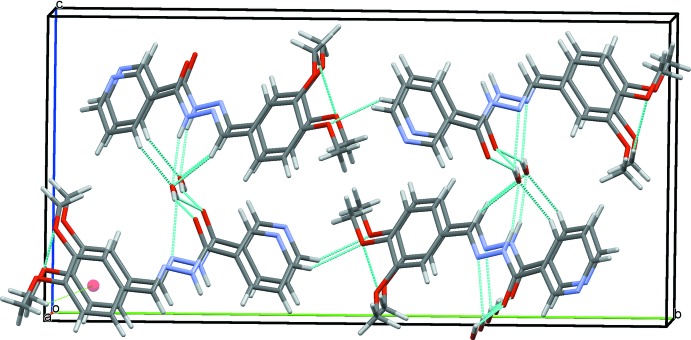
The crystal packing of the title compound viewed along the a axis. Hydrogen bonds are drawn as dashed lines and a representative C–H···π contact is shown as a dotted line.

## supplementary crystallographic information

### S1. Comment

Hydrazones constitute an important class of biologically active drug molecules that have attracted the attention of medicinal chemists due to their wide range of pharmacological properties (Singh & Raghav, 2011). Hydrazone derivatives containing an azomethine (–CONHN═CH–) group have been shown to exhibit antiproliferative activities and act as cytotoxic agents with the ability to prevent cell progression in cancerous cells through different mechanisms (Patil *et al.*, 2011). Moreover, hydrazone derivatives may act as multidentate ligands and their transition metal complexes have been used in the treatment of tuberculosis, in colorimetric or fluorimetric analytic determinations, as well as in applications involving catalytic processes (Torje *et al.*, 2012). As part of our studies of substituent effects on the structure and other aspects of hydrazone derivatives, such as (*E*)—*N*'-(4-Methoxybenzylidene)pyridine-3-carbohydrazide dihydrate (Novina *et al.*, 2013), in the present work we report the synthesis and crystal structure of the title compound.

The molecule of the title hydrazide derivative (Fig. 1), C_15_H_15_N_3_O_3_·H_2_O, exists in a *trans* conformation with respect to the C8═N1 double bond [1.277 (2) Å] with the torsion angle N2—N1—-C8—C5 = -177.58 (14)°. It also adopts the amido form with the C9═O3 bond length of 1.2322 (19) Å which is very close to the reported C═O bond length of a similar structure (Wang *et al.*, 2010). The benzene and pyridine rings (C2—C7 and N3/C10—C14, respectively) are each planar with a dihedral angle of 5.10 (14)° between their mean-planes. This is comparable to the corresponding angle found in a related structure (Novina *et al.*, 2013). One of the methoxy group is almost coplanar with the C2—C7 benzene ring whereas the other one deviates somewhat from the benzene ring plane [torsion angles: C1—O1—C2—C7 = -3.9 (3), C15—O2—C3—C4 = 16.5 (3)°].

The water molecule forms six H–bonds with three different nicotinohydrazone molecules. N—H···O, O—H···O, O—H···N and C—H···O hydrogen bonds are present in the crystal system (Table 1). One of the H atoms of the water molecule forms bifurcated hydrogen bonds to the azomethine nitrogen and the carbonyl oxygen atoms of one neighboring molecule (Fig. 2). The water molecule acts as a hydrogen bond acceptor towards another nicotinohydrazone molecule through N–H···O and C—H···O hydrogen bonds. Through these interactions the molecules are interconnected through the water molecule to form infinite chains parallel to the *b* axis of the unit cell (Fig. 2). Furthermore, a C1—H1C···π interaction involving the phenyl (C2—C7) ring together with O–H···O and C–H···O contacts to generate a three dimensional network of molecules stacked along the *a* axis direction (Fig. 3).

### S2. Experimental

3,4-dimethoxybenzaldehyde (4.1 ml, 0.025 mol) was added to an ethanolic solution of nicotinicacid hydrazide (3.4 g, 0.025 mol). After the addition was complete the reaction mixture was stirred well in an ice cold condition for 3 hrs. The colourless solid that formed was filtered and washed several times with petroleum ether (40–60%). The crude solid obtained was dried and recrystallized from absolute alcohol. The recrystallized product was dried over vacuum.

### S3. Refinement

The H atoms of the solvent water were located in a difference map and refined freely with isotropic displacement parameters with their bond distances restrained to 0.86 (2) Å. Other H atoms were placed in geometrically idealized positions and constrained to ride on their parent atoms with C—H = 0.93 Å, CH_3_ = 0.96 Å, N—H = 0.86 Å and with *U*_iso_(H) = 1.5*U*_eq_(CH_3_) and 1.2*U*_eq_(CH, NH).

### Crystal data

**Table:**

C_15_H_15_N_3_O_3_·H_2_O	*F*(000) = 640
*M**_r_* = 303.32	*D*_x_ = 1.335 Mg m^−^^3^
Monoclinic, *P*2_1_/*n*	Mo *K*α radiation, λ = 0.71073 Å
Hall symbol: -P 2yn	Cell parameters from 2357 reflections
*a* = 4.9128 (6) Å	θ = 4.7–51.8°
*b* = 25.137 (4) Å	µ = 0.10 mm^−^^1^
*c* = 12.2950 (16) Å	*T* = 296 K
β = 96.513 (4)°	Block, colorless
*V* = 1508.6 (4) Å^3^	0.50 × 0.35 × 0.30 mm
*Z* = 4	

### Data collection

**Table:**

Bruker Kappa APEXII CCD diffractometer	3704 independent reflections
Radiation source: fine-focus sealed tube	2250 reflections with *I* > 2σ(*I*)
Graphite monochromator	*R*_int_ = 0.036
ω and φ scan	θ_max_ = 28.3°, θ_min_ = 3.0°
Absorption correction: multi-scan (*SADABS*; Bruker, 2004)	*h* = −6→6
*T*_min_ = 0.952, *T*_max_ = 0.971	*k* = −33→33
11633 measured reflections	*l* = −16→16

### Refinement

**Table:**

Refinement on *F*^2^	Primary atom site location: structure-invariant direct methods
Least-squares matrix: full	Secondary atom site location: difference Fourier map
*R*[*F*^2^ > 2σ(*F*^2^)] = 0.048	Hydrogen site location: inferred from neighbouring sites
*wR*(*F*^2^) = 0.141	H atoms treated by a mixture of independent and constrained refinement
*S* = 1.02	*w* = 1/[σ^2^(*F*_o_^2^) + (0.0655*P*)^2^ + 0.1374*P*] where *P* = (*F*_o_^2^ + 2*F*_c_^2^)/3
3704 reflections	(Δ/σ)_max_ < 0.001
209 parameters	Δρ_max_ = 0.21 e Å^−^^3^
3 restraints	Δρ_min_ = −0.23 e Å^−^^3^

### Special details

**Table:**

Geometry. All e.s.d.'s (except the e.s.d. in the dihedral angle between two l.s. planes) are estimated using the full covariance matrix. The cell e.s.d.'s are taken into account individually in the estimation of e.s.d.'s in distances, angles and torsion angles; correlations between e.s.d.'s in cell parameters are only used when they are defined by crystal symmetry. An approximate (isotropic) treatment of cell e.s.d.'s is used for estimating e.s.d.'s involving l.s. planes.
Refinement. Refinement of *F*^2^ against ALL reflections. The weighted *R*-factor *wR* and goodness of fit *S* are based on *F*^2^, conventional *R*-factors *R* are based on *F*, with *F* set to zero for negative *F*^2^. The threshold expression of *F*^2^ > σ(*F*^2^) is used only for calculating *R*-factors(gt) *etc*. and is not relevant to the choice of reflections for refinement. *R*-factors based on *F*^2^ are statistically about twice as large as those based on *F*, and *R*- factors based on ALL data will be even larger.

### Fractional atomic coordinates and isotropic or equivalent isotropic displacement parameters (Å^2^)

**Table:**

	*x*	*y*	*z*	*U*_iso_*/*U*_eq_	
C1	1.8728 (4)	0.00081 (9)	0.12935 (17)	0.0591 (6)	
H1A	1.7449	−0.0075	0.0667	0.089*	
H1B	1.9736	−0.0306	0.1531	0.089*	
H1C	1.9975	0.0277	0.1101	0.089*	
C2	1.5690 (3)	0.06393 (7)	0.19504 (13)	0.0345 (4)	
C3	1.4107 (3)	0.07841 (7)	0.27937 (13)	0.0349 (4)	
C4	1.2439 (3)	0.12194 (7)	0.26645 (13)	0.0360 (4)	
H4	1.1402	0.1315	0.3221	0.043*	
C5	1.2269 (3)	0.15227 (7)	0.17060 (13)	0.0343 (4)	
C8	1.0486 (3)	0.19839 (7)	0.15615 (13)	0.0376 (4)	
H8	1.0267	0.2158	0.0889	0.045*	
C9	0.6145 (3)	0.27874 (7)	0.28846 (13)	0.0356 (4)	
C10	0.4296 (3)	0.32458 (7)	0.25774 (13)	0.0358 (4)	
C14	0.2785 (4)	0.34525 (9)	0.33488 (15)	0.0512 (5)	
H14	0.2956	0.3293	0.4035	0.061*	
C13	0.0882 (4)	0.40894 (9)	0.22029 (18)	0.0574 (6)	
H13	−0.0306	0.4376	0.2065	0.069*	
C12	0.2313 (5)	0.39213 (11)	0.13952 (19)	0.0764 (8)	
H12	0.2132	0.4093	0.0721	0.092*	
C11	0.4038 (5)	0.34939 (10)	0.15793 (17)	0.0704 (7)	
H11	0.5030	0.3373	0.1028	0.084*	
C6	1.3787 (3)	0.13702 (7)	0.08749 (13)	0.0396 (4)	
H6	1.3658	0.1564	0.0227	0.047*	
C7	1.5492 (3)	0.09318 (7)	0.10011 (13)	0.0388 (4)	
H7	1.6511	0.0834	0.0440	0.047*	
C15	1.2265 (4)	0.05011 (9)	0.43970 (15)	0.0578 (6)	
H15A	1.2328	0.0847	0.4730	0.087*	
H15C	1.2545	0.0234	0.4956	0.087*	
H15B	1.0510	0.0449	0.3982	0.087*	
N1	0.9212 (3)	0.21555 (6)	0.23409 (11)	0.0364 (3)	
N2	0.7497 (3)	0.25839 (6)	0.20898 (10)	0.0370 (3)	
H2N2	0.7300	0.2717	0.1441	0.044*	
N3	0.1091 (4)	0.38654 (8)	0.31852 (15)	0.0637 (5)	
O1	1.7281 (2)	0.01981 (5)	0.21562 (10)	0.0467 (3)	
O1W	1.1763 (3)	0.21759 (7)	0.47728 (11)	0.0606 (4)	
O2	1.4351 (3)	0.04615 (5)	0.36928 (10)	0.0496 (4)	
O3	0.6430 (3)	0.26124 (6)	0.38267 (9)	0.0505 (4)	
H1O1	1.310 (4)	0.2231 (12)	0.440 (2)	0.101 (10)*	
H2O1	1.028 (4)	0.2235 (11)	0.4360 (19)	0.102 (10)*	

### Atomic displacement parameters (Å^2^)

**Table:**

	*U*^11^	*U*^22^	*U*^33^	*U*^12^	*U*^13^	*U*^23^
C1	0.0537 (11)	0.0579 (14)	0.0675 (14)	0.0190 (10)	0.0148 (10)	−0.0139 (11)
C2	0.0304 (8)	0.0321 (9)	0.0414 (9)	−0.0008 (7)	0.0062 (6)	−0.0027 (7)
C3	0.0366 (8)	0.0365 (10)	0.0318 (8)	−0.0006 (7)	0.0052 (6)	0.0023 (7)
C4	0.0401 (9)	0.0401 (10)	0.0291 (8)	0.0058 (8)	0.0094 (6)	−0.0026 (7)
C5	0.0368 (8)	0.0356 (10)	0.0307 (8)	0.0022 (7)	0.0038 (6)	−0.0009 (7)
C8	0.0437 (9)	0.0406 (10)	0.0287 (8)	0.0060 (8)	0.0049 (7)	0.0027 (7)
C9	0.0381 (9)	0.0392 (10)	0.0297 (8)	0.0015 (7)	0.0052 (6)	0.0002 (7)
C10	0.0360 (8)	0.0383 (10)	0.0335 (8)	0.0035 (7)	0.0060 (6)	−0.0004 (7)
C14	0.0625 (12)	0.0551 (13)	0.0375 (10)	0.0177 (10)	0.0117 (8)	0.0020 (9)
C13	0.0603 (12)	0.0493 (13)	0.0627 (13)	0.0206 (10)	0.0075 (10)	0.0025 (10)
C12	0.0972 (17)	0.0813 (18)	0.0549 (13)	0.0482 (15)	0.0267 (12)	0.0252 (12)
C11	0.0879 (16)	0.0809 (18)	0.0476 (12)	0.0467 (14)	0.0305 (11)	0.0188 (11)
C6	0.0448 (9)	0.0441 (11)	0.0310 (8)	0.0003 (8)	0.0094 (7)	0.0046 (8)
C7	0.0391 (9)	0.0432 (11)	0.0368 (9)	0.0017 (8)	0.0152 (7)	−0.0038 (8)
C15	0.0555 (12)	0.0773 (16)	0.0427 (10)	0.0078 (11)	0.0142 (9)	0.0198 (10)
N1	0.0409 (8)	0.0367 (9)	0.0313 (7)	0.0080 (6)	0.0023 (6)	0.0011 (6)
N2	0.0447 (8)	0.0388 (9)	0.0274 (7)	0.0107 (7)	0.0038 (6)	0.0049 (6)
N3	0.0736 (12)	0.0635 (13)	0.0565 (11)	0.0278 (10)	0.0180 (9)	−0.0034 (9)
O1	0.0467 (7)	0.0415 (8)	0.0543 (8)	0.0114 (6)	0.0159 (6)	0.0009 (6)
O1W	0.0661 (10)	0.0840 (12)	0.0303 (7)	−0.0146 (9)	−0.0007 (7)	0.0055 (7)
O2	0.0559 (8)	0.0533 (9)	0.0418 (7)	0.0151 (7)	0.0153 (6)	0.0152 (6)
O3	0.0611 (8)	0.0611 (9)	0.0305 (6)	0.0175 (7)	0.0106 (5)	0.0088 (6)

### Geometric parameters (Å, º)

**Table:**

C1—O1	1.424 (2)	C14—N3	1.331 (2)
C1—H1A	0.9600	C14—H14	0.9300
C1—H1B	0.9600	C13—N3	1.326 (3)
C1—H1C	0.9600	C13—C12	1.348 (3)
C2—O1	1.364 (2)	C13—H13	0.9300
C2—C7	1.373 (2)	C12—C11	1.371 (3)
C2—C3	1.412 (2)	C12—H12	0.9300
C3—O2	1.3652 (19)	C11—H11	0.9300
C3—C4	1.366 (2)	C6—C7	1.382 (2)
C4—C5	1.398 (2)	C6—H6	0.9300
C4—H4	0.9300	C7—H7	0.9300
C5—C6	1.386 (2)	C15—O2	1.418 (2)
C5—C8	1.452 (2)	C15—H15A	0.9600
C8—N1	1.277 (2)	C15—H15C	0.9600
C8—H8	0.9300	C15—H15B	0.9600
C9—O3	1.2322 (19)	N1—N2	1.3806 (19)
C9—N2	1.344 (2)	N2—H2N2	0.8600
C9—C10	1.489 (2)	O1W—H1O1	0.856 (17)
C10—C11	1.370 (3)	O1W—H2O1	0.854 (17)
C10—C14	1.371 (2)		
			
O1—C1—H1A	109.5	N3—C13—C12	123.02 (19)
O1—C1—H1B	109.5	N3—C13—H13	118.5
H1A—C1—H1B	109.5	C12—C13—H13	118.5
O1—C1—H1C	109.5	C13—C12—C11	119.2 (2)
H1A—C1—H1C	109.5	C13—C12—H12	120.4
H1B—C1—H1C	109.5	C11—C12—H12	120.4
O1—C2—C7	125.21 (15)	C10—C11—C12	119.83 (18)
O1—C2—C3	115.16 (15)	C10—C11—H11	120.1
C7—C2—C3	119.62 (15)	C12—C11—H11	120.1
O2—C3—C4	124.52 (14)	C7—C6—C5	120.51 (16)
O2—C3—C2	115.86 (15)	C7—C6—H6	119.7
C4—C3—C2	119.61 (15)	C5—C6—H6	119.7
C3—C4—C5	120.85 (15)	C2—C7—C6	120.38 (15)
C3—C4—H4	119.6	C2—C7—H7	119.8
C5—C4—H4	119.6	C6—C7—H7	119.8
C6—C5—C4	119.01 (16)	O2—C15—H15A	109.5
C6—C5—C8	119.90 (15)	O2—C15—H15C	109.5
C4—C5—C8	121.07 (14)	H15A—C15—H15C	109.5
N1—C8—C5	121.19 (15)	O2—C15—H15B	109.5
N1—C8—H8	119.4	H15A—C15—H15B	109.5
C5—C8—H8	119.4	H15C—C15—H15B	109.5
O3—C9—N2	122.34 (16)	C8—N1—N2	115.72 (14)
O3—C9—C10	121.05 (15)	C9—N2—N1	118.29 (13)
N2—C9—C10	116.61 (14)	C9—N2—H2N2	120.9
C11—C10—C14	116.41 (17)	N1—N2—H2N2	120.9
C11—C10—C9	124.86 (16)	C13—N3—C14	116.83 (17)
C14—C10—C9	118.70 (15)	C2—O1—C1	117.29 (15)
N3—C14—C10	124.68 (18)	H1O1—O1W—H2O1	108 (2)
N3—C14—H14	117.7	C3—O2—C15	116.70 (13)
C10—C14—H14	117.7		
			
O1—C2—C3—O2	−0.8 (2)	C9—C10—C11—C12	−178.5 (2)
C7—C2—C3—O2	177.74 (15)	C13—C12—C11—C10	−0.3 (4)
O1—C2—C3—C4	−179.80 (15)	C4—C5—C6—C7	−1.4 (3)
C7—C2—C3—C4	−1.3 (2)	C8—C5—C6—C7	−179.96 (16)
O2—C3—C4—C5	−178.69 (15)	O1—C2—C7—C6	179.32 (15)
C2—C3—C4—C5	0.2 (3)	C3—C2—C7—C6	1.0 (3)
C3—C4—C5—C6	1.1 (3)	C5—C6—C7—C2	0.4 (3)
C3—C4—C5—C8	179.61 (15)	C5—C8—N1—N2	−177.58 (14)
C6—C5—C8—N1	−175.03 (16)	O3—C9—N2—N1	1.6 (3)
C4—C5—C8—N1	6.5 (3)	C10—C9—N2—N1	−179.42 (14)
O3—C9—C10—C11	174.1 (2)	C8—N1—N2—C9	−178.76 (15)
N2—C9—C10—C11	−4.9 (3)	C12—C13—N3—C14	−0.8 (4)
O3—C9—C10—C14	−3.7 (3)	C10—C14—N3—C13	−0.3 (3)
N2—C9—C10—C14	177.30 (17)	C7—C2—O1—C1	−3.9 (3)
C11—C10—C14—N3	1.0 (3)	C3—C2—O1—C1	174.54 (15)
C9—C10—C14—N3	178.98 (18)	C4—C3—O2—C15	16.5 (3)
N3—C13—C12—C11	1.1 (4)	C2—C3—O2—C15	−162.44 (16)
C14—C10—C11—C12	−0.7 (4)		

### Hydrogen-bond geometry (Å, º)

Cg2 is the centroid of the C2–C7 benzene ring.

**Table:**

*D*—H···*A*	*D*—H	H···*A*	*D*···*A*	*D*—H···*A*
N2—H2*N*2···O1*W*^i^	0.86	2.06	2.8942 (19)	165
O1*W*—H2*O*1···O3	0.85 (2)	2.15 (2)	2.955 (2)	157 (3)
O1*W*—H2*O*1···N1	0.85 (2)	2.49 (2)	3.1087 (19)	130 (2)
C11—H11···O1*W*^i^	0.93	2.30	3.199 (3)	162
C8—H8···O1*W*^i^	0.93	2.67	3.425 (2)	139
O1*W*—H1*O*1···O3^ii^	0.86 (2)	2.09 (2)	2.901 (2)	159 (3)
C1—H1*C*···*Cg*2^ii^	0.96	2.88	3.729 (3)	148

Symmetry codes: (i) *x*−1/2, −*y*+1/2, *z*−1/2; (ii) *x*+1, *y*, *z*.
